# Study protocol for a randomized, double-blind, placebo-controlled trial of a single preoperative steroid dose to prevent nausea and vomiting after thyroidectomy: the tPONV study

**DOI:** 10.1186/1471-2253-13-19

**Published:** 2013-09-09

**Authors:** Ignazio Tarantino, Ulrich Beutner, Walter Kolb, Sascha A Müller, Cornelia Lüthi, Andreas Lüthi, Bruno M Schmied, Thomas Clerici, Rene Warschkow

**Affiliations:** 1 Department of Surgery, Kantonsspital St. Gallen, CH-9007, St. Gallen, Switzerland; 2 Institute of Medical Biometry and Informatics, University of Heidelberg, D-69120, Heidelberg, Germany; 3 Department of Anesthesiology, Kantonsspital St. Gallen, CH-9007, St. Gallen, Switzerland

**Keywords:** Dexamethasone, Thyroidectomy, Prevention, Nausea, Vomiting, PONV

## Abstract

**Background:**

Postoperative nausea and vomiting after general anesthesia is not only an unpleasant problem affecting 20-30% of surgical patients but may also lead to severe postoperative complications. There is a particularly high incidence of postoperative nausea and vomiting following thyroidectomy. Dexamethasone has been described as highly effective against chemotherapy-induced nausea and vomiting and has been proposed as a first-line method of postoperative nausea and vomiting prophylaxis. Despite this possible beneficial effect, the prophylactic administration of dexamethasone before surgery to prevent or ameliorate postoperative nausea and vomiting has not been established. A bilateral superficial cervical plexus block during thyroid surgery under general anesthesia significantly reduces pain. Of even greater clinical importance, this block prevents the need for postoperative opioids. Therefore, patients undergoing thyroidectomy and a bilateral superficial cervical plexus block are an ideal group to investigate the efficacy of dexamethasone for postoperative nausea and vomiting. These patients have a high incidence of postoperative nausea and vomiting and do not require opioids. They have no abdominal surgery, which can cause nausea and vomiting via a paralytic ileus. Combined with the highly standardized anesthesia protocol in use at our institution, this setting allows all known biases to be controlled.

**Methods/design:**

We will perform a parallel two-arm, randomized (1:1), double-blind, placebo-controlled, single-center trial. Adults (≥18 years) scheduled for primary partial or total thyroidectomy because of a benign disease will be eligible for inclusion. The participants will be randomized to receive a single, intravenous preoperative dose of either 8 mg of dexamethasone in 2 ml saline (treatment group) or saline alone (placebo group). All the patients will receive a bilateral superficial cervical plexus block and standardized anesthesia. The primary outcome will be the incidence of postoperative nausea and vomiting. A total of 152 patients will be recruited, providing 80% power to detect a 50% reduction in the incidence of postoperative nausea and vomiting. Any patients who require opioid treatment will be excluded from the per-protocol analysis.

**Discussion:**

In the present protocol, we reduced bias to the greatest extent possible. Thus, we expect to definitively clarify the efficacy of dexamethasone for postoperative nausea and vomiting prophylaxis.

**Trial registration:**

http://www.clinicaltrials.gov: NCT01189292

## Background

### Scientific background and explanation of rationale

Postoperative nausea and vomiting (PONV) is a common and distressing problem that affects 20-30% of surgical patients after general anesthesia [1]. PONV not only reduces patient comfort but can also lead to serious postsurgical complications, such as dehydration, electrolyte imbalances, the aspiration of the gastric contents, esophageal rupture, suture dehiscence and bleeding [2-9]. Furthermore, PONV significantly affects healthcare costs by prolonging hospital stays [10,11]. In particular, patients undergoing thyroidectomy exhibit a high incidence of PONV, which can be as high as 80% [2,12,13]. PONV after thyroidectomy is most likely caused by edema and inflammation around the neck tissues, leading to evoked parasympathetic impulses through the vagus, recurrent laryngeal and glossopharyngeal nerves to the vomiting center [13-15].

Dexamethasone, a glucocorticosteroid, has been described as highly effective against chemotherapy-induced nausea and vomiting [16,17]. A recently published study comparing the efficacy of six well-established antiemetic strategies concluded that the use of dexamethasone as a first-line method of prophylaxis for PONV is a reasonable treatment option [18]. The exact mechanism by which dexamethasone exerts its antiemetic activity is not well understood. The antagonizing effect of corticosteroids on inflammatory reactions and the dexamethasone-triggered release of endorphins, which results in mood elevation, a sense of well-being and appetite stimulation, may contribute to this effect [19-21]. Due to their anti-inflammatory activity, corticosteroids have been shown to reduce postoperative swelling, pain and sore throat [22-25]. Despite these advantages, the perioperative use of dexamethasone for the prophylaxis and treatment of PONV has not been established [26], possibly due to concerns that the use of corticosteroids would increase susceptibility to infections. However, several studies have indicated that even long-term corticosteroids treatments do not lead to a significant increase in wound infections [27-29]. Another possible reason is the lack of awareness about the antiemetic effect of this type of medication.

Recently, a meta-analysis of five studies that investigated the effects of single-dose dexamethasone application prior to thyroidectomy demonstrated a significant reduction in the relative risk of PONV [30]. However, this meta-analysis was based on studies that did not control for factors potentially influencing PONV, such as postoperative opioid administration or the use of different anesthetics. Two of the five included studies enrolled exclusively Asian women [31,32]. Furthermore, serious doubts about the validity of one of the included studies have been expressed [33]. In summary, the generalizability of this meta-analysis is questionable, and further research is warranted to assess the true efficacy of a single, preoperative dose of dexamethasone for PONV prevention following thyroidectomy.

Recently, our group demonstrated that a bilateral superficial cervical plexus block during thyroid surgery under general anesthesia significantly reduced pain. Of even greater clinical importance, this block prevented the need for postoperative opioids [34,35]. Therefore, patients undergoing thyroidectomy and a bilateral superficial cervical plexus block are an ideal, homogenous cohort in which to investigate the efficacy of dexamethasone for postoperative PONV. These patients exhibit a high incidence of PONV and do not require opioids. They have no abdominal surgery that can cause nausea and vomiting via paralytic ileus. Combined with the highly standardized anesthesia protocol in use at our institution, this setting allows all known biases to be controlled.

### Aim of the study

The objective of the present study is to assess the preventive benefit of a single, preoperative dose of dexamethasone for PONV in patients undergoing thyroidectomy without the postoperative administration of opioids. Additionally, the effects on postoperative pain, hospital stay, wound healing and morbidity will be assessed.

## Methods/design

The study was planned according to the updated Consolidated Standards of Reporting Trials (CONSORT) statement [36] and according to the Declaration of Helsinki, the Guidelines of Good Clinical Practice issued by ICH and the requirements of Swiss regulatory authorities.

### Trial design

This study is a single institution, 1:1 randomized, double-blind, placebo-controlled trial with two parallel arms, comparing a single, preoperative dose of 8 mg dexamethasone (treatment group) with saline (placebo group) in a superiority analysis.

### CONSORT diagram

**Figure 1:**
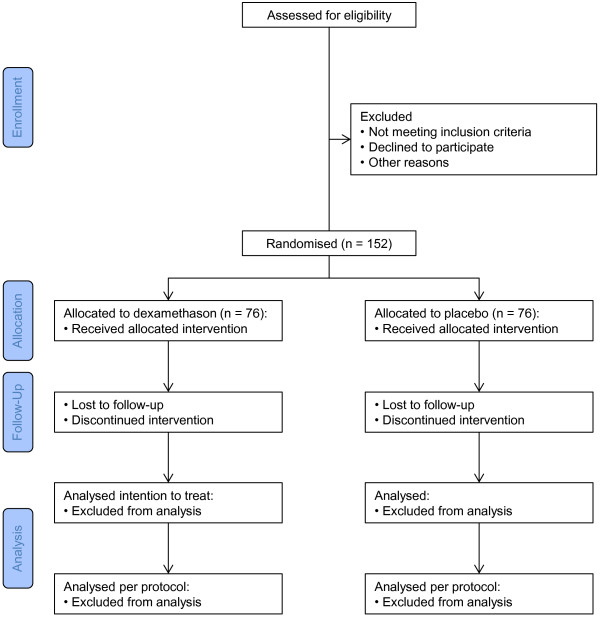
CONSORT flow diagram of the trial.

Figure 1 shows the CONSORT diagram of the trial.

### Eligibility criteria for participants

All the adult patients (aged ≥ 18 years) who are scheduled for a primary partial or total thyroidectomy because of a benign disease at our institution (Kantonsspital St. Gallen, Department of Surgery) will be eligible to participate in the study. The exclusion criteria aim to provide a reliable, balanced and homogeneous study population with high compliance to the study protocol and to avoid adverse effects.“ The inclusion and exclusion criteria are listed below.”

Inclusion criteria:

– Age ≥ 18 years

– Patients scheduled for a partial or total thyroidectomy

Exclusion criteria:

– History of adverse reactions to dexamethasone, propylene glycol or disodium-EDTA

– Chronic pain

– Necessity for sternotomy

– Inability to administer a cervical block (20 ml of 0.5% bupivacaine)

– Diabetes mellitus type 1 or 2 requiring medicamentous treatment

– Pregnancy (tested in all reproductive-age women)

– Glaucoma

– The administration of antiemetic therapy within 48 h before the surgery

– Acute pain requiring analgesics other than acetaminophen or more than a single dose of nonsteroidal anti-inflammatory (NSAID) treatment within 48 h before the surgery

– The use of antipsychotic drugs

– Noncompliance

– Necessity of neck dissection (except compartment C1/level 6)

– Reoperations

### Setting and data collection locations

The patients will be enrolled at the outpatient Clinic of Endocrine Surgery. The participants will be enrolled only by the head of the Clinic of Endocrine Surgery (TC) or under his direct supervision. For each randomized patient, a case report form (CRF) will be generated and updated continuously throughout the hospitalization period. CRFs specifically created for this trial will be used for documentation. All the CRFs are Microsoft Excel-based, and the data will be entered electronically. The CRFs contain extensive probability and range checks to reduce erroneous entries, they are designed to reduce free-text entries to the minimum necessary, and selection from predefined lists (drop-down menus) will be the preferred means of data entry. The completed forms will be printed and signed by the entering physician and then sent to the trial coordinator, along with the data files. The six-week follow-up visits will occur at the outpatient Clinic of Endocrine Surgery.

Several procedures have been designed to enhance the quality of the trial conduct: reviews of the protocol and forms, the direct entry of the data into Excel data entry forms whenever possible, the automatic performance of consistency checks by the electronic data forms, the entry of written or printed data into electronic forms and a review of the data by the trial chair or a delegated person (with the medical content of all the forms reviewed and approved by a monitor).

### Intervention/treatment

Study medication will be prepared by the hospital pharmacy. Medication will be delivered in a syringe containing either 2 ml saline (placebo) or 2 ml saline with 8 mg dexamethasone (Mephameson®, Mepha Pharma, Basel, Switzerland) and are indistinguishable. Syringes will be labeled with the patient’s name, the study name and the study’s patient number.

The anesthesiologist will administer the study medication intravenously 30–60 min before surgery (skin incision) and is completely unaware of the treatment allocation. Thereafter, all the patients will undergo standardized anesthesia, thyroid surgery and routine postoperative care. During the surgery, recurrent laryngeal nerve monitoring will be performed.

A bilateral superficial cervical plexus block will be performed just before the skin incision with a 20-ml syringe and a 20-G × 2^3^/_4_-inch needle (total dose, 20 ml). On each side of the neck, 10 ml of 0.5% bupivacaine solution (Carbostesin® 0.5%; AstraZeneca, Zug, Switzerland) will be administered (total dose, 20 ml) according to the following procedure. On the first side of the neck, along the cranial dorsal edge of the sternocleidomastoid muscle, three deposits of approximately 2.5 ml each will be injected to anesthetize the cervical plexus and its *nervus transversus colli*. To anesthetize the region of the planned skin incision, the remaining 2.5 ml will be injected subcutaneously on each side of the incision. The same procedure will be performed on the opposite side of the neck, using the remaining 10 ml of bupivacaine solution.

The anesthestic technique will be standardized using propofol (Disoprivan® 1%; AstraZeneca) for hypnosis, fentanyl (Sintenyl®, Sintetica, Mendrisio, Switzerland) and remifentanil (Ultiva®;GlaxoSmithKline, Münchenbuchsee, Switzerland) for analgesia, and rocuronium (Esmeron®, MSD, Lucerne, Switzerland) for muscle relaxation.

Propofol will be used as total intravenous anesthesia in a target-controlled infusion (TCI) system according to the Schnider model [37]. Induction will be performed with propofol at an effect-site concentration (Ce) of 4 μg/ml and will be maintained at a Ce of 2–2.5 μg/ml to hold the bispectral index value between 40 and 60. Analgesia will be performed with 0.3-0.4 mg of fentanyl administered intravenously at the beginning of anesthesia. Anaesthesia will be supplemented by remifentanil allowing for stable hemodynamics and avoidance of movements. Remifentanil will be administered in a TCI system according to the Minto model [38], beginning with a Ce of 2–3 ng/ml and increasing to 10–12 ng/ml at the end of anesthesia. Tracheal intubation will be facilitated with intravenous rocuronium at 0.5 mg/kg bodyweight. No further relaxation will be induced because of intraoperative recurrent laryngeal nerve monitoring.

The postoperative first-line therapy for PONV will be intravenous droperidolum (Droperidol®, Sintetica, Mendrisio, Switzerland), with a maximal dose of 3 × 0.5 mg/24 h if the patient’s blood pressure exceeds 120 mmHg. The second-line therapy will be ondansetron (Zofran®, GlaxoSmithKline, Münchenbuchsee, Switzerland) at a dose of 4 mg intravenously.

No routine analgesia is planned. The first-line reserve for pain relief on patient demand will be 1 g of paracetamol (Dafalgan®; Bristol-Myers Squibb, Baar, Switzerland) administered by mouth, with a maximal dose of 3 g/24 h. The second-line reserve will be 1 g of metamizole (Novalgin®; Sanofi-Aventis, Meyrin, Switzerland), with a maximal dose of 4 g/24 h. The third-line reserve will be intravenous morphine (Morphin HCI®, Sintetica, Mendrision, Switzerland); however, its necessity will constitute a protocol violation.

### Outcome measurements

The primary outcome will be the incidence of PONV, which will be assessed at postoperative hours 4, 8, 16, 24, 32 and 48. A patient will be considered to suffer from PONV if nausea or vomiting is documented at any of the postoperative assessments.

One secondary outcome will be the severity of the PONV, measured with a score ranging from 0 to 3 (0 for no nausea; 1 for mild nausea, defined as nausea requiring a single administration of an antiemetic drug; 2 for severe nausea, defined as nausea requiring the repeated administration of antiemetic drugs; and 3 for nausea leading to vomiting).

Other secondary outcomes will be the intensity of pain at rest and under provocation (while rotating the neck 90° to each side), measured at postoperative hours 4, 8, 16, 24, 32 and 48 with a Verbal Rating Scale (VRS) ranging from 0 to 10, with 0 considered to be no pain and 10 considered to be the worst pain imaginable. Additional secondary outcomes will include the length of hospital stay (measured as the number of hours after the intervention), wound healing at the time of discharge and six weeks after the intervention, the amount of anesthetic medication required (total amount of administered fentanyl, remifentanil, rocuronium measured in mg and the total amount of administered remifentanil measured in μg) and postoperative in-hospital morbidity, including wound infection, reoperation, bleeding, prolonged intubation, laryngeal nerve lesion, urinary retention, urinary tract infection, pulmonary infection and gastrointestinal hemorrhage or newly occurring insulin dependence in diabetic patients.

For the safety analysis, adverse events (AEs) and serious adverse events (SAEs) will be evaluated. The patients will be instructed by the investigator to report the occurrence of any AE, defined as an unfavorable and unintended sign, symptom or disease temporally associated with the treatment provided in the present trial. The investigator (treating physician) will be asked to verify the absence or presence of all the AEs predefined on the CRF. Information in the patients’ medical files will be reported using the Common Terminology Criteria for Adverse Events v4.03 (CTCAE 4.03) coding system, rather than a narrative description. AEs will be scored as grade 1 (a mild AE) to grade 5 (death related to the AE), and a causality assessment will be performed.

All grade 4 or 5 AEs will be counted as SAEs, with the exclusion of PONV (as the primary outcome), delayed wound healing (as a secondary outcome) and wound infection (as a secondary outcome). Additionally, postoperative hypoparathyroidism and lesions to the recurrent nerve will not be counted as SAEs because they are considered imminent risks of a thyroidectomy.

The local Ethics Committee and Swissmedic will be informed by the principal investigator about SAEs according to local legislation (VKlin/HMG). A yearly safety summary will be provided to the local Ethics Committee and Swissmedic.

### Sample size calculation

A sample size determination was conducted for the main outcome variable, the incidence of PONV, using R environment version 13.2 with the gsDesign package. A preliminary retrospective analysis of 100 patients revealed a total incidence of PONV of 48% (unpublished data), confirming previous studies [39]. Based on a power of 0.80 (p = 0.05, two-sided) to detect a 50% reduction in the incidence in PONV (48% vs. 24%) as the clinically relevant treatment advantage between two balanced arms, a total of 123 patients were required for a fixed design. To account for deviations from the expected effect size and incidence due to the avoidance of opioids for postoperative pain relief, a group sequential design was implemented with one interim analysis after 70 patients have completed the study treatment. In a symmetric, two-sided group sequential design with an O’Brien-Flemming alpha spending function [40], the inflation factor was 1.006. The adjusted sample size was estimated to be a total of 124 patients (62 patients per arm). With an assumed rate of 18% protocol violations (derived from a preliminary retrospective analysis of 100 patients), the calculated total sample size was 152 patients (76 patients per arm). To recruit this number of patients, a 30-month inclusion period was anticipated.

### Interim analysis

An interim analysis is planned to assess the primary outcome and the safety of the treatment after 70 patients. For the interim analysis, the data manager will provide the data to the trial statistician with coded treatment assignments (A, B). The statistician will provide summary tables of grade 3 and 4 SAEs and a blind analysis of the primary outcome. Based on these data, the trial chairperson and co-chairperson will decide whether to continue the trial without modifications, continue the trial with modifications or halt the trial due to safety or efficacy concerns. Only if the results are insufficient for that decision the treatment assignments will be revealed and the data will be analyzed again. Unless the benefit of the treatment is demonstrated “beyond a reasonable doubt” by the interim analysis, no formal discontinuation due to efficacy is foreseen. The decision about trial continuation will be based not only on the statistical results but also on clinical judgment [41]. The statistical boundary, requiring p < 0.0039 at the interim analysis to halt due to efficacy, will serve as a guide. The results of the interim analysis will be confidential and strictly limited to the trial statistician, trial chairperson and trial co-chairperson. To avoid bias in the subsequent trial, the results of the interim analysis will be treated confidentially and will not be communicated to the outside or to the clinical investigators involved in the trial.

### Randomization: sequence generation, allocation concealment mechanism and implementation

The randomization will be 1:1 for each arm without stratification. Treatment allocation will be performed according to predefined block randomization list with random block sizes between four and ten. Only the data manager (UB) has access to this computer generated randomization list. Two to three days before surgery the data manager will prepare a computer generated study medication request form based on the patient’s study number. The request contains the patient’s name and the treatment allocation. The form will be placed in an opaque, sealed and signed envelope and will be directly delivered to the pharmacy by study personnel. Study medication will be prepared according to the request form by the hospital pharmacy as described earlier and will be delivered to the anesthesiologist by an assigned surgical resident.

If the allocated study treatment planned cannot be performed, the patient will remain on the study and will be included in the intention-to-treat (ITT) analysis. An explanation for the non-adherence must be supplied.

In case of an emergency two principal investigators (TC, IT) and the data manager can break the blinding of single patients. Unblinding is computer-based and is only available if the principal investigators are signed-in as users. Before unblinding a reason for unblinding has to be given. For each unblinding, the reason, the requestor, the computer ID, the time and date are recorded. The data manager will be automatically informed in case of an unblinding.

### Statistical analysis

The statistical analysis will be performed with SAS 9.1 (SAS Institute Inc., Cary, NC, USA). Missing values will be replaced with the last available value (the last observation carried forward approach, LOCF). For the baseline characteristics, descriptive statistics will be used as appropriate.

Two analyses (interims analysis and final analysis) will be performed for the primary outcome. To maintain the alpha, an O’Brien-Flemming alpha spending function [40] will be used. The alpha values used for the primary outcome will be 0.0039 in the interim analysis and 0.0488 in the final analysis. Both alphas will be adjusted for the exact proportion of information included in the interim analysis if the analysis is not performed for exactly 70 patients. For all other analyses, two-sided significance tests with an alpha of 0.05 will be applied.

All confirmatory analyses will be performed primarily as ITT analyses; the available data from all the patients will be assessed for the outcomes according to the randomization. To assess the treatment effects of dexamethasone vs. placebo in patients with no postoperative use of opioids, additional analyses will be conducted excluding the patients with protocol violations.

For the superiority analysis of dexamethasone vs. placebo in terms of the incidence of PONV (the primary outcome), a chi-square statistic will be applied. Mixed models assessing the mean ranks of the scores with adjustments for time and treatment vs. time interactions will be applied to analyze the following secondary outcomes: the severity of PONV, the intensity of pain at rest and the intensity of pain under provocation. The Mann–Whitney U statistic will be used to assess the following secondary outcomes: the required amount of anesthetic medication, the amount and type of postoperatively administered analgesics and the length of hospital stay. Postoperative morbidity and wound healing at the time of discharge and six weeks after the intervention will be assessed using a chi-square statistic.

Auxiliary, non-confirmatory analyses will be performed to assess the influences of baseline and treatment characteristics on the primary and secondary outcomes.

### Blinding

The patients, all personnel involved in patient care or treatment, the data collectors, and the outcome adjudicators will be blind to the treatment allocation. Only the data manager of the trial, who is not involved in the treatment or patient care, has access to the treatment allocations. For the interim analysis the statistician will be provided with non-descriptive arm assignments (A, B). If the trial chairperson or co-chairperson decides that the occurrence of AEs or SAEs necessitates un-blinding, the data manager of the trial will reveal the treatment allocation of that patient.

### Ethical issues

This protocol, the patient information sheet and the patient consent form have been reviewed and approved by the local Ethics Committee of the Canton St. Gallen (EKSG10/082/2B) and by Swissmedic (2011DR3005) prior to enrolling any patients in this trial. All the patients will be informed about the aims and procedures of the trial, possible adverse events, how to react if an adverse event occurs and possible hazards to which they may be exposed. The participants will be informed that their patient data will be held strictly confidential but that that their medical records may be reviewed for trial purposes by authorized individuals other than their treating physician. It will be emphasized that participation is voluntary and that the patients are allowed to refuse further participation in the trial whenever they want. Data obtained prior to the withdrawal of the patient will be included in the analysis. Written informed consent will be required for all the patients entering the trial. Two copies of the consent form must be signed, one of which will be retained by the patient. The clinical investigator who enters the patient into the trial is responsible for obtaining informed consent.

## Discussion

The present study investigating the efficacy of a single, preoperative dose of dexamethasone for PONV in patients undergoing thyroidectomy will be the first in the literature to explicitly control for all known sources of bias. The setting proposed in the present protocol is ideal for several reasons. First, patients undergoing thyroidectomy have a high incidence of PONV [2,12,13]. Therefore, these patients are ideal subjects for testing new antiemetic strategies because the study will unlikely be underpowered and the patients will not be unnecessarily exposed to the inherent risks. Second, the included patients do not undergo abdominal surgery, which can cause a postoperative paralytic ileus that can also provoke nausea and vomiting [42]. In such a confounding setting, accurately differentiating between the nausea and vomiting caused by the anesthesia and that caused by the operation itself would be impossible. Third, through the application of the cervical block, postoperative opioids should not be needed by the majority of the patients [35]. As well-known causes of nausea and vomiting, opioids might also confound the analysis of the efficacy of dexamethasone for preventing PONV [42]. Although prohibiting the use of opioids in the present study would have been ideal, it is not possible for ethical reasons. Therefore, opioid administration, if necessary, is defined as an exclusion criterion. A difficulty arises from the allocation of patients to a specific arm (active drug vs. placebo) prior to any decision to administer the opioid medication; therefore, patients receiving opioids cannot be excluded from the ITT analysis. As a consequence, the per-protocol analysis will be critically important for evaluating the true efficacy of dexamethasone in preventing PONV. Fourth, all the patients will receive the same standardized anesthesia protocol, which will be crucial for avoiding sources of bias that were not considered in previous studies [30].

We would like to acknowledge the potential limitations of this study protocol. First, although the scale for PONV measurement has been previously used it is not an explicitly validated scale. Nevertheless, as only patients vomiting or receiving antiemetic medication will be considered to suffer PONV the bias resulting from subjective ratings should be minimal. Second, recent studies suggested that 5-HT3 receptor antagonists might be more effective than dexamethasone in preventing postoperative nausea and vomiting [43]. Nevertheless, in times of constant pressure on health care expenses, the low cost and the general availability of dexamethasone still make it a relevant option for prevention of PONV. Third, although no opioids are applied in the postoperative course they are unavoidable during general anesthesia. Thus it could be possible that differences in operation time might lead to a different amount of applied remifentanil, potentially leading to a bias in PONV. As the study is randomized we consider such a bias very unlikely. However, since we record the amount of applied fentanyl and remifentanil, in case of an imbalance we will be able to adjust for this bias. Fourth, for pain measurement we will use a Verbal Rating Scale ranging from 0 to 10 instead of a 100 mm Visual Analog Scale. At our institution the use of this Verbal Rating Scale for pain measurement during the postoperative course is a common and well established clinical routine. Furthermore, patients are preoperatively informed to rate their pain using the Verbal Rating Scale.

In the present project, we reduced bias as much as possible. Thus, we expect to definitively clarify the efficacy of dexamethasone for PONV prophylaxis. The confirmation of this efficacy by our study will have a major impact because the results may establish dexamethasone administration before thyroidectomy as a new standard in clinical practice. Moreover, the results of this study could be applicable to all types of operations that involve general anesthesia and will stimulate further clinical research in this area.

### Trial status

The trial began in October 2011, and recruitment is ongoing.

## Abbreviations

CONSORT: Consolidated standards of reporting trials; CRF: Case report form; Ce: Effect-site concentration; PONV: Postoperative nausea and vomiting; AE: Adverse event; SAE: Serious adverse event; LOCF: Last observation carried forward; TCI: Target-controlled infusion; VRS: Verbal rating scale; CTCAE: Common terminology criteria for adverse events.

## Competing interests

The authors declare that they have no competing interests.

## Authors’ contributions

RW, TC, WK and IT developed the original study design. RW, TC, SAM and IT developed the research protocols. RW and IT performed the sample size calculation and a preliminary retrospective study to assess the incidence of PONV. SAM, BMS, TC and IT are responsible for the clinical input. RW, IT and UB drafted the manuscript. CL performed the preliminary retrospective study to assess the incidence of PONV. All the authors have approved the final manuscript.

## Pre-publication history

The pre-publication history for this paper can be accessed here:

http://www.biomedcentral.com/1471-2253/13/19/prepub
